# Cocaine Poisoning

**Published:** 1892-11

**Authors:** J. D. Warford

**Affiliations:** Linwood, Kan.


### Cocaine Poisoning.

I notice a case of cocaine poisoning, reported in the *Satellite* for May, by Dr. J. A. Wessinger, of Ann Arbor, Mich.

I have recently had a similar experience.

Was called, March 1st, to see Mrs. M., who was suffering from malignant trouble of right breast. I at once decided to remove the growth. She objected to the use of chloroform, as she had taken it once before when, as she said, it caused some heart trouble.

I, therefore, decided to use cocaine. Having prepared a fresh solution, I proceeded to inject it into the subcutaneous tissues surrounding the tumor.

She was almost immediately seized with a severe nervous rigor or chill. The surface became cool and blue; lips pale and thin; respiration very slow and sighing; pulse 150 to 160 and irregular; with almost complete unconsciousness.

I injected brandy, and digitalis hypodermically, with hot applications, and within an hour's time reaction was fully established.

I then gave her a general tonic treatment, and on March 17th I resorted to chloroform and performed the operation, the patient making a good recovery.

I have been using cocaine almost recklessly for the last four or five years, and this is the first unpleasant symptom I ever met from its use. Was it the cocaine, or was it nervous shock?

I am a great admirer of cocaine—I use it often and use it freely as a local anæsthetic, and have heretofore regarded it as a harmless remedy.

It is the most satisfactory remedy for the painless (?) extraction of teeth I ever used; and for operations on the eye it has no equal.

J. D. WARFORD, M.D.

Linwood, Kan.

### Salicylic Acid.

To increase the solubility of salicylic acid in water the addition of one part of acid to one hundred parts of glycerine and one hundred and fifty parts of water gives, it appears, the best results. This mixture is clear and miscible with water without any alteration.

### Dental Neuralgia.

For neuralgias of dental origin, Prof. Da Costa said that gelsemium is especially useful. Begin treatment by giving five drops of the tincture three times a day, and increase to ten drops three times a day until the patient sees double, and then stop the administration of the drug.

